# Leptin receptor co-expression gene network moderates the effect of early life adversity on eating behavior in children

**DOI:** 10.1038/s42003-022-03992-8

**Published:** 2022-10-14

**Authors:** Randriely Merscher Sobreira de Lima, Barbara Barth, Danusa Mar Arcego, Euclides José de Mendonça Filho, Sachin Patel, Zihan Wang, Irina Pokhvisneva, Carine Parent, Robert D. Levitan, Michael S. Kobor, Ana Paula Santana de Vasconcellos Bittencourt, Michael J. Meaney, Carla Dalmaz, Patrícia Pelufo Silveira

**Affiliations:** 1 Department of Psychiatry, Faculty of Medicine, McGill University, Montreal, QC Canada; 2 Programa de Pós-Graduação em Neurociências, Instituto de Ciências Básicas da Saúde (ICBS), Universidade Federal do Rio Grande do Sul, Porto Alegre, RS Brazil; 3 Integrated Program in Neuroscience (IPN), McGill University, Montreal, QC Canada; 4 Programa de Pós-Graduação em Psicologia, Instituto de Psicologia, Universidade Federal do Rio Grande do Sul, Porto Alegre, RS Brazil; 5 Ludmer Centre for Neuroinformatics and Mental Health, Douglas Research Centre, McGill University, Montreal, QC Canada; 6 Department of Psychiatry, University of Toronto and Centre for Addiction and Mental Health, 250 College St, Toronto, Ontario Canada; 7 Centre for Molecular Medicine and Therapeutics, BC Children’s Hospital Research Institute, Department of Medical Genetics, The University of British Columbia, 938 West 28th Avenue, Vancouver, BC Canada; 8 Departamento de Ciências Fisiológicas, Universidade Federal do Espírito Santo, Espírito Santo, Brazil; 9 Singapore Institute for Clinical Sciences, Agency for Science, Technology and Research (A*STAR), Singapore, Singapore; 10 Programa de Pós-graduação em Ciências Biológicas: Bioquímica, Departamento de Bioquímica, Instituto de Ciências Básicas da Saúde, Universidade Federal do Rio Grande do Sul, Porto Alegre, RS Brazil

**Keywords:** Feeding behaviour, Stress and resilience

## Abstract

Leptin influences eating behavior. Exposure to early adversity is associated with eating behaviour disorders and metabolic syndrome, but the role of the leptin receptor on this relationship is poorly explored. We investigated whether individual differences in brain region specific leptin receptor (LepR) gene networks could moderate the effects of early adversity on eating behavior and metabolism. We created an expression-based polygenic risk score (ePRS) reflecting variations in the function of LepR gene network in prefrontal cortex and hypothalamus to investigate the interactions between a cumulative index of postnatal adversity on eating behavior in two independent birth cohorts (MAVAN and GUSTO). To explore whether variations in the prefrontal cortex or hypothalamic genetic scores could be associated with metabolic measurements, we also assessed the relationship between LepR-ePRS and fasting blood glucose and leptin levels in a third independent cohort (ALSPAC). We identified significant interaction effects between postnatal adversity and prefrontal-based LepR-ePRS on the Child Eating Behavior Questionnaire scores. In MAVAN, we observed a significant interaction effect on food enjoyment at 48 months (*β* = 61.58, *p* = 0.015) and 72 months (*β* = 97.78, *p* = 0.001); food responsiveness at 48 months (*β* = 83.79, *p* = 0.009) satiety at 48 months (*β* = −43.63, *p* = 0.047). Similar results were observed in the GUSTO cohort, with a significant interaction effect on food enjoyment (*β* = 30.48, *p* = 0.006) food fussiness score (*β* = −24.07, *p* = 0.02) and satiety score at 60 months (*β* = −17.00, *p* = 0.037). No effects were found when focusing on the hypothalamus-based LepR-ePRS on eating behavior in MAVAN and GUSTO cohorts, and there was no effect of hypothalamus and prefrontal cortex based ePRSs on metabolic measures in ALSPAC. Our study indicated that exposure to postnatal adversity interacts with prefrontal cortex LepR-ePRS to moderate eating behavior, suggesting a neurobiological mechanism associated with the development of eating behavior problems in response to early adversity. The knowledge of these mechanisms may guide the understanding of eating patterns associated with risk for obesity in response to fluctuations in stress exposure early in life.

## Abstract (web summary)

An expression-based polygenic risk score analysis of leptin receptor (LepR) genes suggests that LepR-specific genes co-expressed in the prefrontal cortex interact with exposure to postnatal adversity, potentially modulating eating behavior in children.

## Introduction

Genes and environment interact to influence resilience and susceptibility to metabolic disorders^1^. Adverse events occurring early in life are related to distinct phenotypes of eating behavior and overeating, and increased risk for several metabolic diseases during development^2^. Studies with animal models evaluating the effects of neonatal stress on feeding behavior and body weight regulation have observed that early life stress exposure affects food consumption and eating choices^3^, and leptin moderates this relationship^4^.

Leptin is an adipose-tissue-derived hormone involved in physiological processes that include appetite, body weight regulation and emotional behavior^5^. Leptin also plays an important role in the regulation of glucose metabolism^6^. Leptin crosses the blood-brain barrier, and its effects on feeding behavior are mediated by receptors located in the arcuate nucleus of the hypothalamus (HPT), piriform cortex and hindbrain^7,8^. In humans and rodents, leptin receptor mRNA has also been identified in the hippocampus, striatum, amygdala and prefrontal cortex (PFC)^9^. The non-hypothalamic aspect of the leptin network is related to a diversity of functions, not only food intake, but also motivation, learning, memory, cognitive function, and neuroprotection^10^. In addition to physiological and homeostatic regulations, eating behavior is modulated by hedonic and prefrontal decision-making processes, that have been highly associated with satiety^11^.

Genetic variations can trigger changes in gene expression or functionality, with associated phenotypic variation and disease susceptibility. Mutations in the leptin receptor gene, for example, have been associated with obesity and pituitary dysfunction^12^. Several studies are focused on candidate polymorphisms and their biological effects. However, common diseases can include dysfunctions at many levels, including in genes, cells or brain regions, as well as on the feedback between these structures at multiple biological scales^13^. The analysis of genomic data using a co-expression network of genes that interact on functional pathways allows the investigation of connections with biological mechanisms, integrating genomic data and associated phenotypes^14^.

Early environment stress can lead to changes in eating behavior and metabolism, and the leptin receptor is tightly involved in these outcomes through its action on the hypothalamus and prefrontal cortex. Our hypothesis is that being exposed to stress during a sensitive period of life induces changes in leptin signaling, which alters eating behavior and metabolism. Therefore, we aimed to evaluate whether genetic variations that reflect individual differences in gene expression of brain-specific LepR gene network moderate the effects of adversity on eating patterns in children. For this, we constructed expression-based polygenic risk scores that reflect the function of prefrontal cortex or hypothalamus LepR gene networks, based on external mice expression data, and analyzed the effect of their interaction with postnatal adversity on eating behavior of healthy children in different samples. We used the polygenic risk scores to explore variations in metabolic measurements associated with the leptin receptor gene network. We also performed functional enrichment analysis to characterize the biological processes associated with the leptin receptor gene network.

## Results

### Interaction effects between leptin receptor expression-based polygenic risk score and environmental adversity on eating behavior

**Fig. 1:**
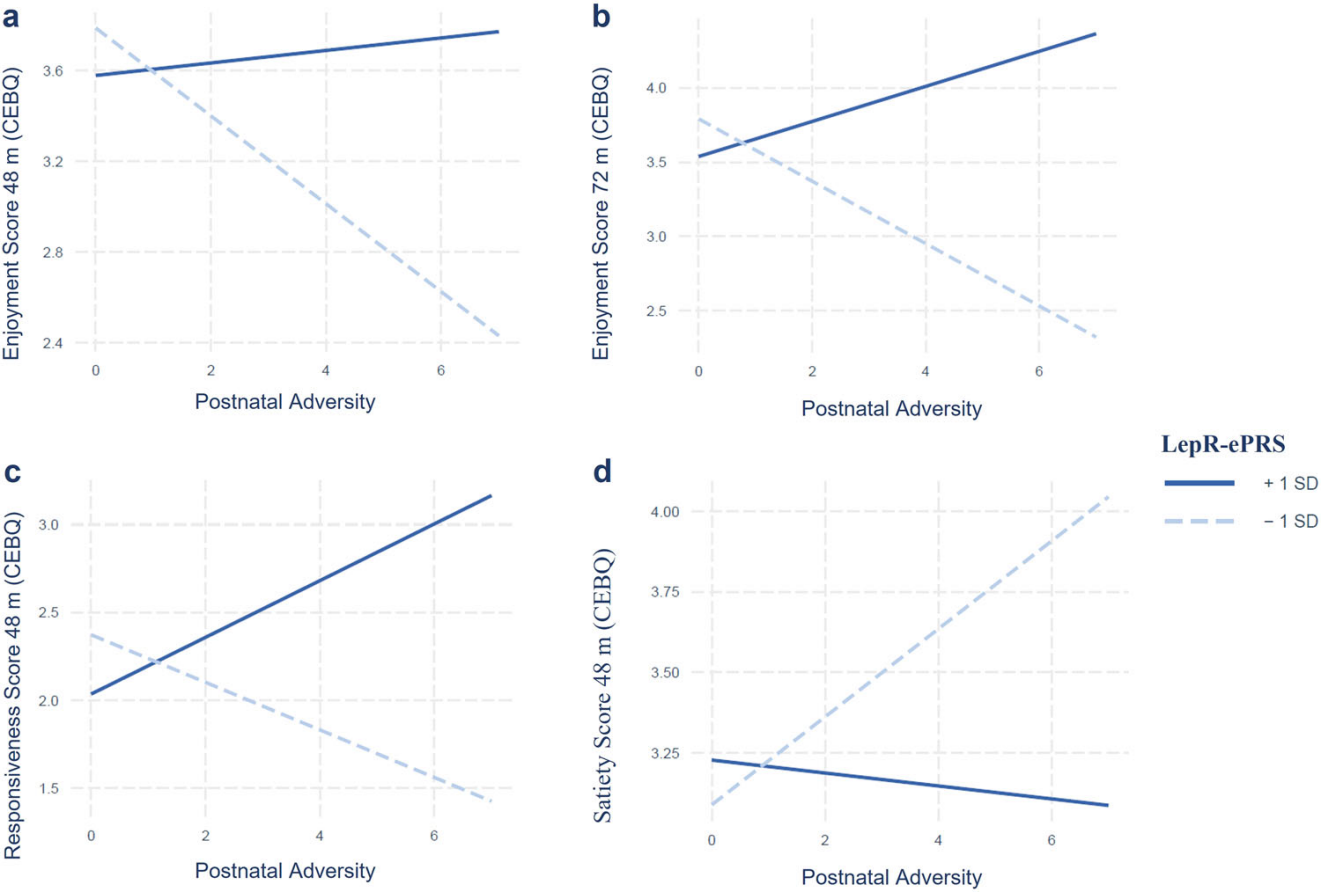
Interaction effect between the Postnatal Adversity Score and Prefrontal-based LepR-ePRS in the MAVAN cohort. Interaction effect between the Postnatal Adversity Score and Prefrontal-based LepR-ePRS on: **a**) Enjoyment score at 48 and **b**) 72 months, **c**) Food responsiveness score at 48 months and **d**) Satiety score at 48 months. Increased postnatal adversity exposure is associated with higher satiety and lower food enjoyment as the ePRS decreases, and with higher Food responsiveness as the ePRS increases. MAVAN cohort (48 m N = 133, 72 m N = 129). Linear regression analyses followed by simple slope analyses.

In MAVAN cohort, we observed statistically significant interaction effects between the adversity score and the prefrontal cortex-based LepR-ePRS on the following domains of the Child Eating Behavior Questionnaire (CEBQ): food enjoyment score at 48 months (*β* = 61.58, *p* = 0.015; CI 12.71─110.44) and 72 months (*β* = 97.78, *p* = 0.001; CI 39.4─156.17); food responsiveness score at 48 months (*β* = 83.79, *p* = 0.009; CI 21.6─146.01), but not 72 months (*p* > 0.05); satiety score at 48 months (*β* = −43.63, *p* = 0.047; CI −86.22 to −1.04) but not 72 months (*β* = −41.15, *p* = 0.093; CI −88.91─6.6) (Fig. 1). We also observed a significant main effect of the PFC LepR-ePRS on slowness in eating score at 48 months (*β* = −71.89, *p* = 0.026; CI −134.90 to −8.89) and a trend at 72 months (*β* = −60.92; *p* = 0.09; CI −132.03─10.18) (Supplementary Fig. 1). No significant main effects or interactions were found on the desire to drink score, emotional over and under eating scores, food fussiness at 48 and 72 months (*p* > 0.05). Illustrative simple slope analysis using ePRS at mean ± SD showed that increased postnatal adversity exposure is associated with higher satiety (48 months *β* = 0.13, *p* = 0.01) and poorer food enjoyment (48 months: *β* = −0.19, *p* = 0.0001; 72 months: *β* = −0.20, *p* = 0.002) as the LepR-ePRS score decreases, and higher food responsiveness as the LepR-ePRS score increases (48 months *β* = 0.16, *p* = 0.02). After correction for multiple testing using Bonferroni-Holm method the interaction analysis on food enjoyment score at 72 months remained significant.

**Fig. 2:**
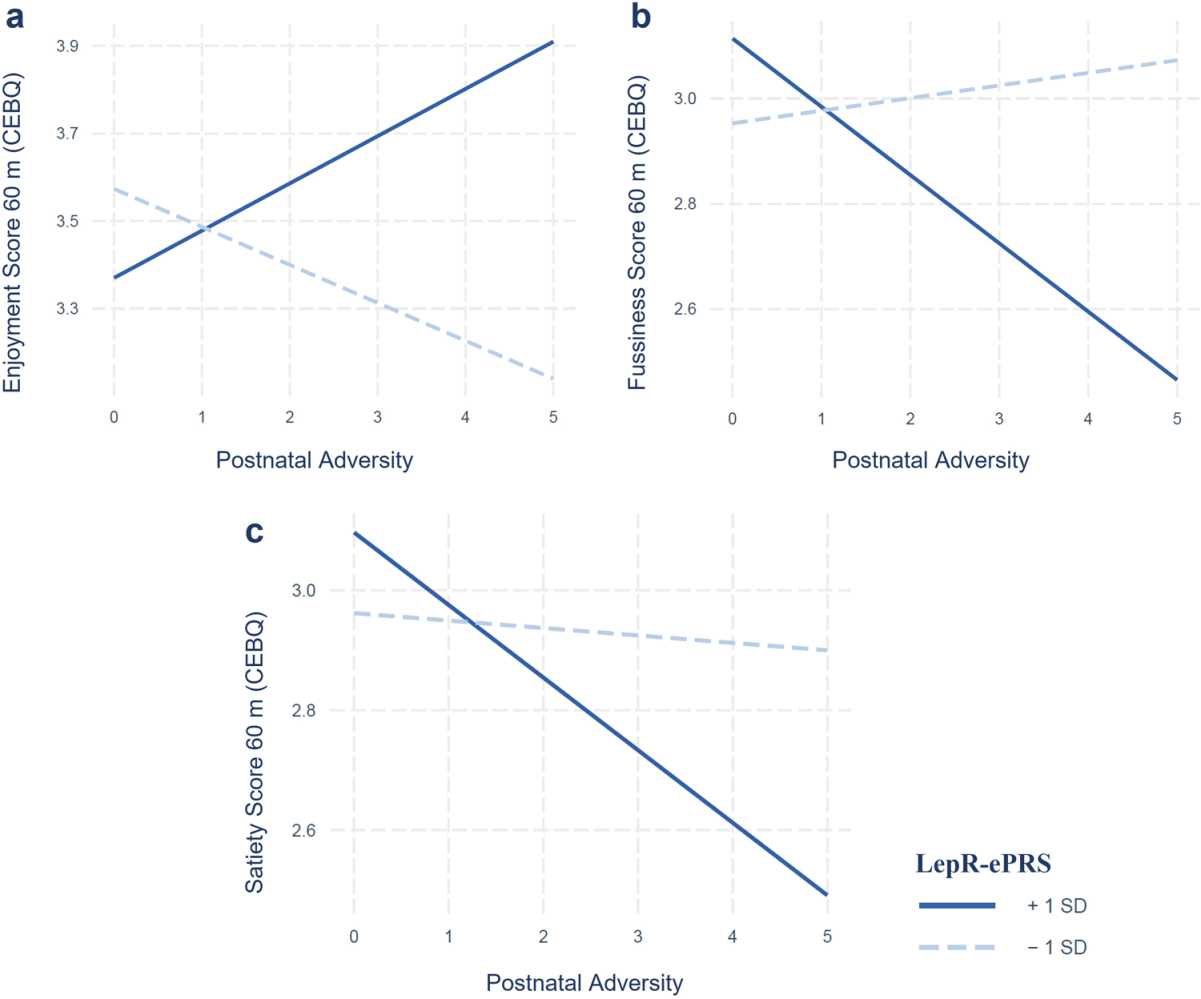
Interaction effect between the Postnatal Adversity Score and the Prefrontal-based LepR-ePRS in the GUSTO cohort. Interaction effect between the Postnatal Adversity Score and the Prefrontal-based LepR-ePRS on **a**) Enjoyment score, **b**) Food fussiness score, and **c**) Satiety score at 60 months in the GUSTO sample. Higher postnatal adversity exposure is associated with lower satiety, higher food enjoyment and lower food fussiness as the ePRS increases. GUSTO cohort (*N* = 439). Linear regression analyses followed by simple slope analyses.

In the GUSTO cohort similar results were found. We observed significant interaction effects between adversity exposure and the prefrontal cortex-based LepR-ePRS on food enjoyment (60 months *β* = 30.48, *p* = 0.006; CI 8.65─52.32), food fussiness score (60 months *β* = −24.07, *p* = 0.02; CI −44.37 to −3.77) and satiety score (60 months *β* = −17.00, *p* = 0.037; CI −32.97 to −1.03). No interaction effects were found in the other domains analyzed, and no main effect of the prefrontal cortex-based LepR-ePRS was observed (*p* > 0.05). A simple slope analysis using ePRS at mean ± SD showed that increased postnatal adversity exposure is associated with lower satiety responsiveness (*β* = −0.12, *p* = 0.0006), as well as with higher enjoyment (*β* = 0.10, *p* = 0.02) and lower fussiness (*β* = −0.12, *p* = 0.003) as the LepR-ePRS score increases (Fig. 2).

No significant main effect of the HPT LepR-ePRS or interaction effect between the HPT LepR-ePRS and adversity were found on any domains of the CEBQ in either the MAVAN or GUSTO cohorts (*p* > 0.05, Supplementary Tables 1 and 2), suggesting that the effects of the LepR gene network in response to adversity are specific for the genes composing this network in the PFC.

To analyze whether variations in the PFC or hypothalamic genetic scores are associated with variations in circulating leptin and glucose levels, we used ALSPAC sample. No significant effects of these ePRSs were found on circulating leptin in children at 9.5 years and fasting glucose measurements in children at 8.5 years (PFC LepR-ePRS [Leptin *β* = 19.53, *p* = 0.16, *N* = 3544; Fasting glucose *β* = 13.80, *p* = 0.40, *N* = 688]); HPT LepR-ePRS [Leptin *β* = −13.93, *p* = 0.23, *N* = 3544; Fasting glucose *β* = −12.45, *p* = 0.35, *N* = 688]).

### LepR co-expression networks

**Fig. 3:**
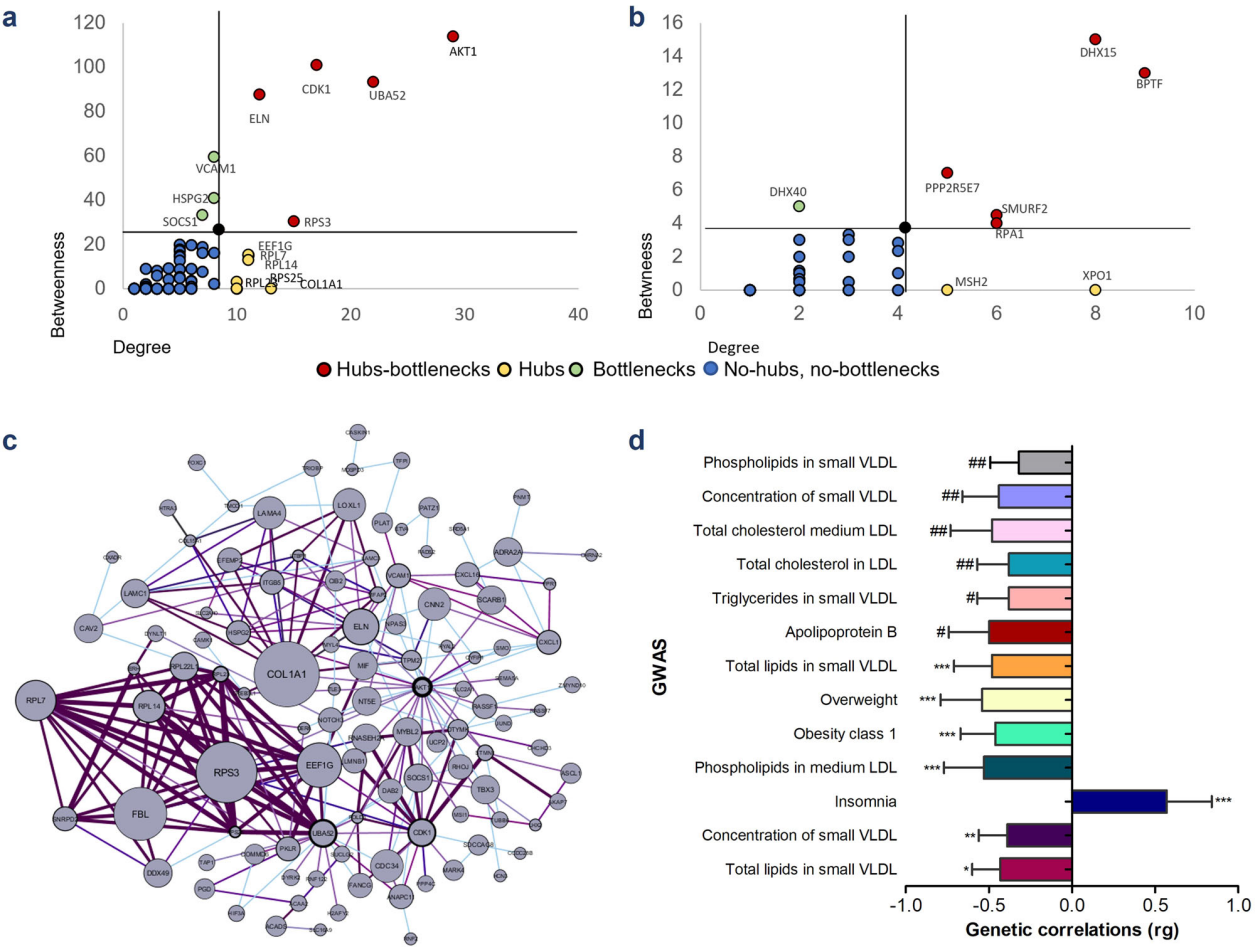
Leptin receptor gene network. Topological properties of proteins belonging to the LepR-ePRS in the PFC (**a**) and HPT (**b**), showing hubs (with degrees higher than 1 SD above the mean), bottlenecks (betweenness higher than 1 SD above the mean), and hub-bottlenecks. Lines in black indicate mean + 1 SD for degrees and betweenness. **c** Node size represents the InDegree (connections between the protein with other proteins); Node border thickness represents OutDegre (connections of other proteins with the target protein); Edge thickness represents co-expression between genes. **d** Genetic correlations (rg) of different traits and diseases associated with SNPs from LepR-ePRS in the prefrontal cortex. Results from LD SC Broad Institute^55^. Error bars indicate the standard error (SE) of rg. *p ≤ 0.01, **p ≤ 0.02, ***p ≤ 0.03, #*p* ≤ 0.04, ##*p* ≤ 0.05.

The Leptin Receptor co-expression network in the PFC was composed by 175 genes, and the network in the HPT was composed by 109 genes. The Cytoscape Software® was used to determine how the genes aggregate in a network (Fig. 3c and Supplementary Fig. 2) and to identify the nodes that could be considered hubs and bottlenecks (Fig. 3a, b). We observed, using String-db, that the gene network in the PFC had more significant protein-protein interactions than in the HPT (PFC: *p* = 6.24e-06; HPT: *p* = 1.62e-05).

To better understand the biological functions of the LepR gene network on the prefrontal cortex, we performed several enrichment analyses. MetaCore® (Clarivate Analytics) showed statistically significant gene ontology processes associated with the ePRS score. In the PFC network, biological processes were enriched for nervous system development (FDR *q* = 4.44e-09), response to lipids (FDR *q* = 9.32e-08), anatomical structure morphogenesis (FDR *q* = 1.63e-08), response to nutrient levels (FDR *q* = 4.72e-08), among others (Supplementary Table 3). Cell type-specific enrichment showed that genes from the PFC LepR network are overexpressed in the amygdala, cortex, and thalamus during early developmental stages (Supplementary Fig. 3). Using PANTHER enrichment analyses, we observed that the PFC LepR gene network is associated with binding, and catalytic activity function regulation (Supplementary Fig. 3). The LepR network in the PFC was also enriched for synaptic components (Supplementary Fig. 3).

Genetic correlations between the SNPs used to create the LepR-ePRS and several GWASes related to lipid metabolism and obesity were found in LD-hub using the PFC scores (Fig. 3d). These results suggest that the PFC LepR network is highly associated with increased risk to the development of metabolic and endocrine disorders. Although our behavioral data were analyzed in childhood, the interaction of this network with adversities in early life can predict children who are vulnerable to emotional eating disorders before the onset of metabolic diseases.

## Discussion

We demonstrated that a biologically-informed polygenic score based on genes co-expressed with the leptin receptor in the PFC moderates the association between postnatal adversity and eating behavior patterns. These effects were seen at different ages and in two independent birth cohorts. No significant interactions or main effects were observed using the hypothalamus-based LepR-ePRS, suggesting a brain-region specificity for the LepR gene network in interacting with the environment to predict eating behavior.

The CEBQ was designed to identify different aspects of eating behavior that have been hypothesized to contribute to body weight regulation^15^. We observed in MAVAN a decrease in food enjoyment and responsivity to food when facing an increase in adversity exposure as the PFC LepR-ePRS decreases. In the GUSTO cohort, we observed that increased postnatal adversity exposure is also related to variations in food enjoyment, with increases in food enjoyment as adversity exposure increases being moderated by LepR-ePRS scores, replicating the results of the MAVAN cohort. Although the significant slope is different between the two cohorts, the interaction seems to tease apart a group of children that responds to adversity with obesogenic behaviors (higher LepR-ePRS score) and another group that responds to adversity with a more anorexic pattern (lower LepR-ePRS score). These two domains, food responsiveness and food enjoyment, reflect eating in response to environmental food cues^16^. Appetite and eating rate are increased in overweight or obese children^16,17^. Stress exposure increases the appetite for sweet foods, and studies with animals have reported that the type, duration or severity of stress may modify responses to stress^18^. Stress also has an effect on food choices. Most stressed individuals show preferences for palatable and calorie-enriched foods^19^. In animal models, chronic stress leads to increased ingestion of sweet food^20^. Previous work has established that leptin acts as a moderator of the relationship between stress exposure and altered eating behavior, possibly due to its action in the brain circuits related to hedonic and homeostatic controls of appetite^11^. Animal studies of stress exposure and food intake suggest that the effect of stress exposure on body weight gain and food intake is mediated by a reduction in NPY^3^, a peptide regulated by peripheral leptin and ghrelin^5^. In humans, Tomiyama and colleagues have demonstrated that, although humans and animals eat palatable foods after stress, an immediate higher leptin response to a stressor was related to less consumption of both high fat and high sugar food^4^. Our study supports previous findings and highlights how a whole network of genes co-expressed with the leptin receptor in the PFC interacts with environmental adversity to influence eating behavior.

Satiety responsiveness was affected by the interaction between the LepR gene network and environmental adversity in both cohorts and at different ages. Satiety represents the ability of a child to reduce food intake after eating, to regulate energy intake. Infants tend to be highly responsive to internal hunger and satiety cues, whereas this level of responsiveness decreases with advancing age^16^. Thus, children will gradually lose the ability to effectively self-regulate energy intake^21^ as we observed in satiety outcomes measured at 72 months. Once again, when comparing the two cohorts we see that, despite the fact that the statistically significant simple slope is different in the two samples, the interaction is able to identify a group of children that respond to adversity with less satiety (higher LepR-ePRS score) and another group that responds to adversity with more satiety (lower LepR-ePRS score). Our study contributes to both identifying and characterizing phenotypes associated with vulnerability to obesogenic and anorexic tendencies when facing adversity in children.

Food fussiness is usually defined as rejection of a substantial amount of familiar foods as well as novel foods, thus leading to the consumption of an inadequate variety of foods. This type of eating behavior is characterized by a lack of interest in food, and slowness in eating^22^. The interaction between exposure to adversity and the LepR-ePRS also modulated fussiness behavior, and this result was observed mainly in the GUSTO cohort. Additionally, we observed an ePRS main effect on slowness to eat, with a lower ePRS being associated with greater slowness to eat, which corroborates the other results found in both cohorts. High scores on the slowness in eating scale are characterized by a reduction in eating rate as a consequence of a lack of enjoyment and interest in food. Obese children show increased food consumption, with less of a reduction in the eating rate towards the end of a meal^22^. However, it is important to note that this effect was not associated with response to adversity.

Provided that leptin is an essential regulator of homeostasis^23^, improving insulin sensitivity and reducing lipolysis through its action on adipocytes^24^, we wanted to explore if the ePRS scores representing the prefrontal and hypothalamic leptin gene networks would be associated with variations in peripheral metabolic markers, using the ALSPAC cohort. Our data demonstrated that fasting glucose and leptin did not vary according to variations in these gene networks, suggesting that the reported effects on eating behavior are likely due to specific effects of the leptin receptor gene network in the central nervous system, and not through modulation of the peripheral metabolism. It is well known that leptin plays a role in the regulation of glucose metabolism and homeostasis, independently of its effect on energy balance^6^. Variations in the LepR-ePRS in the PFC or HPT did not associate with any changes in peripheral metabolic biomarkers, hence the changes seen with regards to eating behavior must be due to the central action of the PFC LepR gene network.

Energy balance is a process regulated by the central nervous system and peripheral organs. The gastrointestinal tract is responsible for the digestion and absorption of nutrients but also sends information to the brain about the perception of gastrointestinal fullness and satiety^25^. Adipose tissue releases hormones that act in the brain to regulate feeding behavior and body weight. The hypothalamus detects and integrates the metabolic signals from fat stores, stimulating or inhibiting food consumption according to energy balance^10^. However, eating behavior is stimulated not only by hunger, but also by hedonic sensations associated with feeding behavior, as well as motivation and inhibitory/decision-making processes. Knowing that the PFC is an important region involved in the modulation of these processes^26^, the results found in this study may be associated with the modulation of motivation to feed through the central action of genes co-expressed with leptin in this region. The hypothalamus is more involved in the control of food intake and homeostasis, and the HPT-based gene network used here may be less able to moderate the influence of adversity on food behavior, given the importance of hypothalamic processes for survival. In addition, the LepR gene network created in the hypothalamus was not filtered by genes overexpressed early in life, which could affect the outcomes analyzed, since postnatal adversity may influence specifically the genes overexpressed in the developing brain at the time when adversity is occurring. Also, the HPT network of genes co-expressed with LepR was a less integrated network when compared to the PFC network.

Evidence suggests that food consumption and eating choices in response to stress exposure are moderated by circulating leptin^4,27^. For example, higher serum leptin concentrations are associated with a decrease in snack intake following a stress situation^28^, suggesting that leptin is associated with greater palatability for snacks, as observed in our results in the food responsiveness score. In addition, leptin modulates brain networks that influence reward and motivation^6,7^. Much of the motivation to obtain food occurs by the activation of the limbic system, prefrontal cortex and ventral tegmental area^11^. The mesolimbic dopaminergic system is the most involved in the control of pleasure and reward^11^. Studies indicate that the regulation of the dopaminergic system in the prefrontal cortex is related to the risk for obesity, being mediated by the D2 receptor^26^. Pathways between the hypothalamus and the mesolimbic system form a functional network for food control. Leptin acts directly on this system, suppressing food consumption and modulating the activity of dopaminergic neurons^29^.

The two brain regions analyzed here have different roles in regulating eating behaviors, consequently the two genetic scores also represent very specific gene networks. *DYRK2* (dual-specificity tyrosine-(Y)-phosphorylation regulated kinase 2) is the only common gene between the PFC and HPT-based genetic scores. We validated our LepR network using several approaches, especially by demonstrating its brain-region specificity. The ePRS method is a reliable approach that goes beyond finding an association between genetic variants and phenotypes^2^. Gene coexpression networks offer information on a functional genomic scale, and have the potential to highlight molecular mechanisms associated with specific behaviors in response to environmental variation. Interestingly, one of the hub genes in the PFC LepR gene network is *AKT1*. This gene is one of three closely related serine/threonine-protein kinases (*AKT1*, *AKT2*, and *AKT3*) called *AKT* kinase, that regulates many processes including metabolism, proliferation, cell survival, growth and angiogenesis^30^. Leptin activates the *PI3K/AKT* pathway, and inhibition of this pathway in the brain prevents the induction of leptin anorexia^30,31^. The presence of this gene in the leptin receptor gene network demonstrates the importance of analyzing genes co-expressed with the target gene, since much of the effects of the network are modulated by hub genes.

It is valuable to note some limitations in our study. First, its important to keep in mind that Genotype-Tissue Expression (GTEx), used to calculate the ePRS, is based mostly on Caucasian participants, and therefore the effect size of the association between alleles and gene expression, as well as allele frequencies, may be different in other ancestries. In that sense, results seen in GUSTO, a Singapore based cohort, may not be as accurate as in the Caucasian samples. Despite this limitation, findings in GUSTO were aligned with the findings observed in MAVAN. Additionally, we observed in the MAVAN cohort that the interactions between the LepR-ePRS and the adversity score are more noticeable at 48 months of age. Evidence shows that during childhood, children experience a critical period for the development of behavioral control of food intake, progressively establishing dietary intake patterns, eating habits, and food preferences^32,33^. Therefore, it can be challenging to identify differences in the same scales throughout development, although some tendencies can be observed. Finally, the CEBQ is a parent-based questionnaire^15^, and several individual characteristics, such as culture and eating habits, could influence the parent’s perception of children’s eating behavior. In our study, we included cohorts of very different food cultures. Nonetheless, the interaction between LepR-ePRS and the postnatal adversity score influencing the eating behavior of children could be observed in both cohorts.

Our data supports the hypothesis that the PFC leptin receptor gene network moderates the effects of postnatal adversity on eating behavior. The findings show the possibility of exploring how different brain gene networks involved with leptin signaling and their interactions with the environment influence the neurobiological basis of eating. The knowledge of these mechanisms may help us to understand eating patterns associated with risk for obesity in response to fluctuations in stress exposure early in life, and inform on preventive and therapeutic practices.

## Methods

### Study design and participants

We used data from the Maternal Adversity, Vulnerability and Neurodevelopment Project (MAVAN), a prospective birth cohort, that followed children at different time points in the first years of life in Montreal (Quebec), and Hamilton (Ontario), Canada^34^. Eligibility criteria for mothers included aged 18 years or above, singleton gestation, and fluency in French or English. Exclusion criteria included severe maternal chronic illness, placenta previa, and history of incompetent cervix, impending delivery, or a fetus/infant affected by a major anomaly or born at a gestational age less than 37 weeks and birth weight <2000g. Birth records were obtained directly from the birthing units. Approval for the MAVAN project was obtained from obstetricians performing deliveries at the study hospitals and by the ethics committees and university affiliates (McGill University and Université de Montréal, the Royal Victoria Hospital, Jewish General Hospital, Centre hospitalier de l’Université de Montréal and Hôpital Maisonneuve-Rosemont) and St Joseph’s Hospital and McMaster University, Hamilton, Ontario, Canada. Informed consent was obtained from all participants^34^.

The Growing Up in Singapore Towards healthy Outcomes (GUSTO) cohort^35^ is based in Singapore and was used as a replication cohort. In GUSTO, pregnant women aged 18 years and above were recruited at the National University Hospital (NUH) and KK Women’s and Children’s Hospital (KKH), being of Chinese, Malay or Indian ethnicity, with homogeneous parental ethnic background. Mothers receiving chemotherapy, psychotropic drugs or who had type I diabetes mellitus were excluded. Informed written consent was obtained from each participant^35^.

The Avon Longitudinal Study of Parents and Children (ALSPAC)^36,37^ was used for additional analyses. The ALSPAC is a cohort that recruited pregnant women living in the county of Avon in the United Kingdom^36,37^. This cohort was used to analyze the effect of LepR-ePRS on leptin and fasting glucose in children aged 9.5 and 8.5 years. All pregnant women (*N* = 14,541 + *n* = 913 enrolled at later phases, *N* = 15,454 total) residing in that part of the old administrative county of Avon were eligible to participate in ALSPAC if the estimated delivery date (EDD) fell between 1 April 1991 and 31 December 1992. Ethical approval of the ALSPAC study was obtained from the Ethics and Law Committee and Local Research Ethics Committees. A full list of the ethics committees that approved different aspects of the ALSPAC studies is available at http://www.bristol.ac.uk/alspac/researchers/research-ethics/. Consent for the use of biological samples was collected in accordance with the Human Tissue Act (2004). From the pregnancy index, over a period of about 20 years, women and children were followed up with questionnaires and assessments of metabolic, cognitive and psychological functions. Data were collected during clinic visits or with postal questionnaires. The ALSPAC study website contains details of all the data that is available through a fully searchable data dictionary and variable search tool available at: http://www.bristol.ac.uk/alspac/researchers/our-data/. Informed consent for the use of data collected via questionnaires and clinics was obtained from participants following the recommendations of the ALSPAC Ethics and Law Committee at the time.

### Genotyping

In MAVAN, autosomal SNPs were genotyped using genome-wide platforms (PsychArray/PsychChip, Illumina) according to manufacturer’s guidelines with 200 ng of genomic DNA derived from buccal epithelial cells. SNPs with a low call rate (<95%), low *p*-values on Hardy–Weinberg Equilibrium (HWE) exact test (p < 1e-40), and minor allele frequency (MAF < 5%) were removed, leaving 242,211 SNPs after the QC procedure. Afterward, imputation using the Sanger Imputation Service^38^ and the Haplotype Reference Consortium (HRC) as the reference panel (release 1.1) was performed and autosomal SNPs with an info score >0.80 were retained for the analysis, resulting in 20,790,893 SNPs. Quality control procedure was carried out using PLINK 1.951^39^. Samples with a call rate less than 90% were removed.

In GUSTO, SNPs were genotyped using the Illumina OmniExpress + exome array. The data was split on three set by ethnicity and quality control procedure was carried out separately on each subset. Non-autosomal SNPs, SNPs with call rates <95%, or minor allele frequency <5%, or those that failed Hardy–Weinberg Equilibrium were excluded from the analysis. Variants discordant with their respective subpopulation in the 1000 Genomes Project^40^ reference panel were removed (Chinese: EAS with a threshold of 0.20; Malays: EAS with a threshold of 0.30; Indian: SAS with a threshold of 0.20). Samples with call rate <99%, cryptic relatedness and sex/ ethnic discrepancies were excluded. The resulting data were pre-phased using SHAPEIT v2.837 with family trio information. We then used Sanger Imputation Service for imputation, choosing 1000 Genomes Project Phase 3 as reference panel and imputed “with PBWT, no pre-phasing” (the Positional Burrows Wheeler Transform algorithm) as the pipeline. Imputed data that were non-monomorphic, had biallelic SNPs and an INFO score > 0.80 were retained. Imputed genotyping data that were common in all three ethnicities (5,771,259 SNPs) were used for further analyses.

Children in ALSPAC were genotyped using the Illumina HumanHap550 quad genome-wide SNP genotyping platform (Illumina Inc., San Diego, CA, US) by the Wellcome Trust Sanger Institute (Cambridge, UK) and the Laboratory Corporation of America (Burlington, NC, US)^41^. The following quality control procedure was applied: participants with inconsistencies in self-reported and genotyped sex, minimal or extreme heterozygosity, high levels of individual missingness (>3%), and insufficient sample replication (IBD < 0.8) were excluded. SNPs with a MAF of <1%, a call rate of <95%, or those not in HWE (*p* < 5 × 10^−7^) were removed. Imputation was conducted using Impute v3 and Haplotype Reference Consortium (HRC) imputation reference panel (release 1.1). The resulting data set consisted of 38,898,739 SNPs available for analysis.

### Procedures

**Fig. 4:**
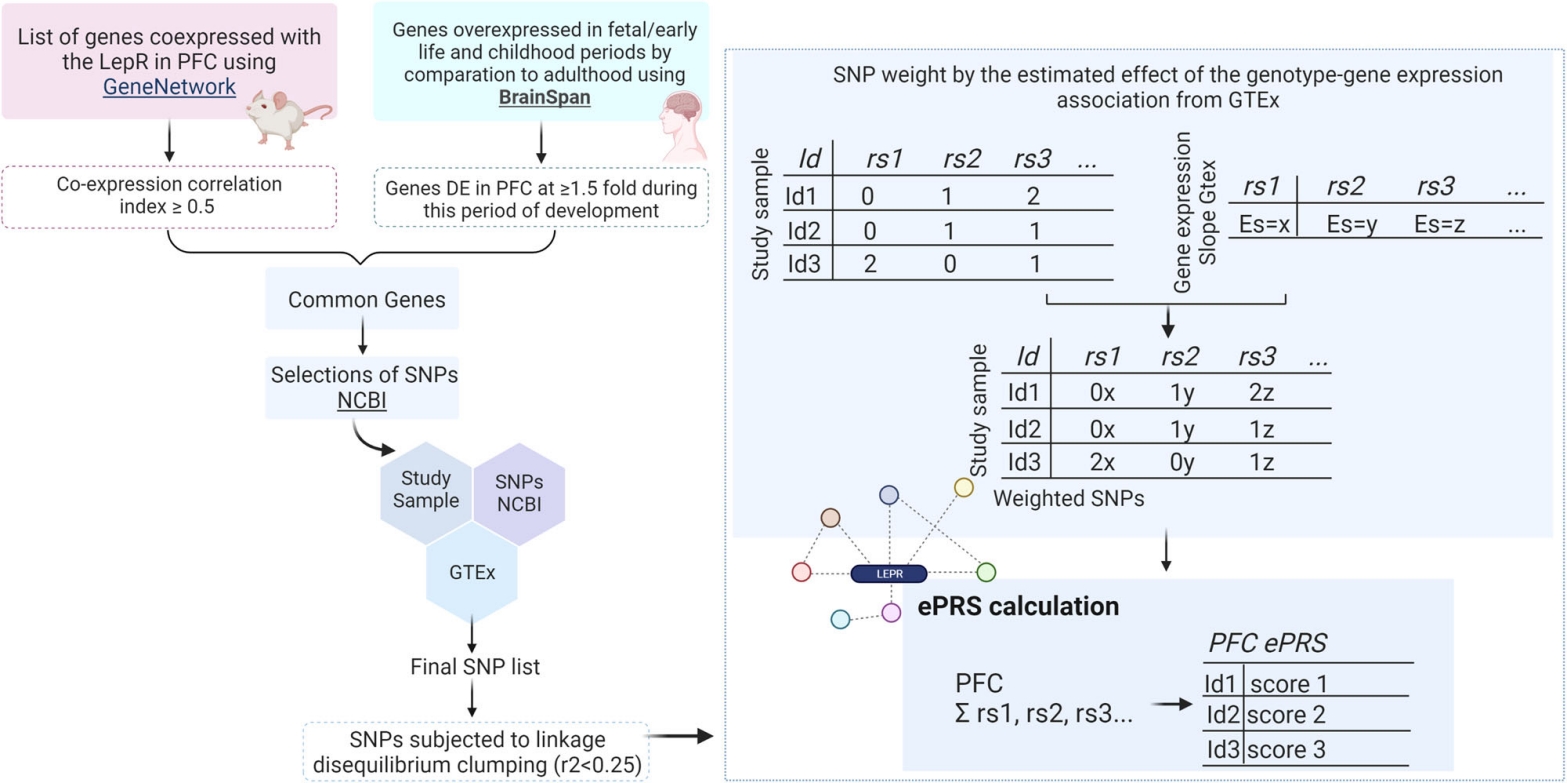
Schematic presentation of the steps involved in the creation of the expression-based polygenic risk score based on genes co-expressed with the leptin receptor (LepR-ePRS). Schematic presentation of the steps involved in the creation the expression-based polygenic risk score based on genes co-expressed with the leptin receptor using the prefrontal cortex (PFC) based LepR-ePRS as an example. Genetic score for the leptin receptor gene network in the PFC: GeneNetwork database was used to generate a list of genes co-expressed with LepR in the PFC in mice. Then mice genes were converted to human orthologs. BrainSpan database was used to identify genes from this list with enrichment within the human PFC during early development. Since we were interested in gene networks that were active during early developmental periods, we retained only the genes overexpressed in early life in comparison to adulthood. Then, we gathered all the SNPs from these genes using the National Center for Biotechnology Information, and merged this list with SNPs from the GTEx data in human PFC. The list of common SNPs was subjected to linkage disequilibrium clumping (r^2^ < 0.25). Our final list of genes included 175 genes. The SNPs were weighted by the SNP-gene expression association slope from the GTEx project. Alleles at a given cis-SNP were weighted by the estimated effect of the genotype associated with gene expression (expression quantitative trait loci from GTEx, in which the effect allele is the alternative allele). The summation of these values from the total number of SNPs provides the ePRS-LepR score.

The expression-based polygenic risk score was created for the prefrontal cortex and the hypothalamus, considering genes co-expressed with the leptin receptor gene (LepR-ePRS), according to the protocol described by Silveira et al.^2,42,43^. The genetic score for the leptin receptor gene network in the prefrontal cortex was created using different tools. GeneNetwork (http://genenetwork.org) was used to generate a list of genes co-expressed with LepR in the PFC in mice. Only genes with an absolute value of the co-expression correlation index higher or equal to 0.5 were kept. Direction of the co-expression was accounted for in ePRS. The list of genes was converted to human genes and then, the BrainSpan (http://www.brainspan.org) was used to identify transcripts from this list with enrichment within the human PFC during early development. Since we were interested in gene networks that were active during early developmental periods (when adversity occurred), we retained only the genes overexpressed in early life in comparison to adulthood (25 weeks gestation to 18 months postnatal in comparison to 20–40 year old adults), selecting genes that were differentially expressed in PFC at ≥1.5 fold during this period of development^44^. Based on their functional annotation in the National Center for Biotechnology Information, U.S. National Library of Medicine (NCBI Variation Viewer), using GRCh37.p13, we gathered all the existing SNPs from these genes, and merged this list with SNPs from the GTEx data in human PFC. The list of common SNPs was subjected to linkage disequilibrium clumping (*r*^2^ < 0.25). Alleles at a given cis-SNP were weighted by the estimated effect of the genotype associated with gene expression (expression quantitative trait loci from GTEx, in which the effect allele is the alternative allele). For more information, see Hari Dass et al., 2019^45^. The summation of these values from the total number of SNPs provides the PFC ePRS-LepR score. Please see Fig. 4. Our final list of genes included 175 genes (Supplementary Data 1). Each ePRS was constructed separately for each study cohort, including all the SNPs existent in the gene network and according to SNPs available in that sample.

The genetic score for the leptin receptor gene network in the hypothalamus was created using the GeneNetwork (http://genenetwork.org) to identify the genes co-expressed with LepR in the HPT in mice (correlation r higher or equal to 0.5 were kept). The list of genes was converted to human genes. Since BrainSpan has no information about the hypothalamus, we gathered all the existing SNPs from these genes, and merged this list with SNPs from the GTEx data in the human HPT. The list of common SNPs was subjected to linkage disequilibrium clumping (*r*^2^ < 0.25), the SNPs were weighted by the SNP-gene expression association slope from the Genotype-Tissue Expression project, data specific to the hypothalamus. Alleles at a given cis-SNP were weighted by the estimated effect of the genotype associated with gene expression (expression quantitative trait loci from GTEx, in which the effect allele is the alternative allele). The final list of genes for the HPT ePRS-LepR score included 109 genes (Supplementary Data 2). The complete list of SNPs used to calculate the ePRS in each cohort can be found in Supplementary Data 3. The PFC LepR-ePRS consisted of 2569 SNPs in MAVAN and 1442 SNPs in GUSTO cohorts and the HPT LepR-ePRS had 1574 SNPs in MAVAN and 679 SNPs in GUSTO, respectively. Figure 4 illustrates the steps involved in the creation of the ePRS for both brain regions.

To validate the gene network used to create the LepR-ePRS, we performed several enrichment analyses using the entire human genome as the background gene set. We used the MetaCore® (https://portal.genego.com) to investigate gene ontology processes, sets as provided by GeneGO (relevant genes in the human genome)^46^. STRING database^47^ was used to performed analyze functional interactions between the proteins from our list LepR-ePRS, co-expressed genes. We used the STRING data to visualize the LepR-ePRS, co-expressed gene network using Cytoscape Software®^48^, Numerical data at supplementary Data 4 (Fig. 3). The topological properties of the gene network were also calculated using the Cytoscape Software® data. Degrees are calculated by summing the number of adjacent nodes, giving information about the local topology, while betweenness considers the number of shortest paths linking two nodes and passing through a node n. Nodes with degrees higher than + 1 SD above the mean were considered hubs, and nodes with betweenness higher than + 1 SD above the mean were considered bottlenecks, and were identified as central nodes within the network^49–51^. Gene Ontology was also accessed using the PANTHER tool^52^, using a whole genome function view. Cell Type-Specific Expression Analysis tool (CSEA)^53^ was used to the identify the localization of genes co-expressed with the LepR-ePRS gene network in human brain regions throughout development. We finally used the SynGO tool^54^ (Synaptic Gene Ontologies and annotations) the to characterize synaptic process associated with the Leptin receptor gene network. LD score regression was used to investigate genetic correlations between SNPs used to create the LepR-ePRS and SNPs associated with diseases or traits through LD Hub^55,56^.

### Postnatal adversity score

The postnatal adversity score was created using a measure combining multiple indicators of adversity, and the presence of each component yielded 1 point (Table 1). The total score represents the summation of all points. The instruments used as indicators of postnatal adversity in MAVAN cohort included:

**Table 1:** Variables and their cut-offs used to create the Postnatal Adversity score in MAVAN and GUSTO.

Postnatal Adversity Score	
MAVAN	GUSTO
- Hospitalization in the first 6 months of life	- Hospitalization in the first 6 months of life
- Birth size percentile below 10th percentile or above 90th percentile	- Birth size percentile below 10th percentile or above 90th percentile
- Gestational age below or equal to 37 weeks	- Gestational age below or equal to 37 weeks
- Maternal mental health (presence of either BDI above 14, EPDS above 9 or STAI above 92)	- Maternal mental health (presence of either BDI above 13, EPDS equal to or above 12 or STAI above 92)
- Household total gross income below 30.000$	- Household monthly income below SG$2000
- Lack of money score above 7	
- Disorganized attachment	
- Poor family function (score greater or equal to 2.00)	- Poor family function (FAD score greater or equal to 2.17)
- Presence of domestic violence or sexual abuse	
- Marital strain score above 3.32	
- Smoking during pregnancy	- Smoking during pregnancy
- Pregnancy anxiety greater than 1.95	

Presence of each component (described in each bullet) yielded 1 point, and the score represents a summation of the points.

**Health and well-being questionnaire**: This questionnaire is a composite of validated short versions of many measures^57^: (a) A subscale from the Daily Hassles was used to measure how often, and to what degree, the woman has lacked money for basic needs (food, heating and electricity) since the beginning of pregnancy^58^. (b) The Marital Strain Scale of Pearlin and Schooler was used to assess chronic stress with the romantic partner. The nine items represent the three items with the highest factor loadings for each of the three factors contributing to the measure of marital strain^59^. (c) The Abuse Assessment Screen was used to assess conjugal violence. This five-item instrument assesses the frequency, severity, perpetrator, and body sites of injury^60,61^. (d) Questions about anxiety during pregnancy^62,63^.

**Smoking during pregnancy:** Composed of yes or no questions;

**Household gross income**: according to ref. ^64^;

**Birth weight and gestational age**: birth records were obtained directly from the birthing unit. Birth weight percentiles were calculated using the local ref. ^65^. **Child Health Questionnaire:** Includes questions on acute and chronic conditions, as well as hospitalizations^66^. **Maternal mental health:** information was extracted from different questionnaires. Beck Depression Inventory, is a 21-question multiple-choice self-report inventory, one of the most widely used psychometric tests for measuring the severity of depression^67^. Edinburgh Postnatal Depression Scale (EPDS), a 10-item self-report scale designed to screen for postpartum depression^68^. State-Trait Anxiety Inventory (STAI), a self-report questionnaire that consists of two forms of 20 items each to measure psychic components of state and trait anxiety^69^.

**Attachment:** The Preschool Separation – Reunion Procedure (PSRP) was applied at 36 months. The PSRP is a modified and developmentally appropriate version of the Ainsworth Strange Situation used to measure attachment security in preschool-aged children^70^. The task consists of a baseline interaction, followed by two separation and reunion episodes lasting 5 min; scoring was based on video coding (reliability *k* = 0.83). Four categories were assessed: secure, ambivalent, avoidant and disorganized attachment. **Family Assessment Device**: a 60-item self-report instrument developed to assess the six dimensions of family functioning outlined in the McMaster Model of Family Functioning^71^. The first six scales assess problem solving, communication, roles, affective responsiveness, affective involvement and behavior control. A general functioning scale assesses overall health-pathology^72^. In GUSTO, the tools were also similar, although there was no information on attachment, domestic violence, lack of money, pregnancy anxiety, and marital strains.

### Outcomes

The Child Eating Behavior Questionnaire (CEBQ)^15^ was used to evaluate the child’s eating behavior. This questionnaire has eight scales and thirty-five questions related to eating styles and satiety. Domains include Desire to Drink, Satiety Responsiveness, Emotional overeating, Emotional undereating, Enjoyment of Food, Food Responsiveness, Slowness in Eating and Food Fussiness^15^. Parents of 143 children at the age of 48 and 72 months (MAVAN, main cohort) and 467 at the age of 60 months (GUSTO, replication cohort) were answered the CEBQ questions.

To explore whether variations in the PFC or hypothalamic genetic scores could be associated with variations in circulating leptin and glucose levels measurements we used the ALSPAC cohort. The Before Breakfast Study in ALSPAC was used to access leptin and fasting glucose from children aged 9.5 and 8.5 years. Plasma glucose was measured using a glucose oxidase test (Y.S.I., Lynchford House, Farnborough, Hants, UK)^73^. Non fasting blood samples from children were collected and samples spun immediately and frozen at −80 °C. Leptin levels were measured by an in‐house ELISA validated against commercial methods^74,75^.

### Statistics and reproducibility

Data were analyzed using R (version 4.1.1). Two-sided significance levels for all measures were set at *P* < 0.05, and confidence intervals (CI) were reported at 95%. Linear regressions were used to examine the effects of interactions between the polygenic scores with the adversity score on the behavioral outcomes (CEBQ). Sex and population stratification principal components were included as covariates. Simple slope analyses were conducted to identify post-hoc differences for the statistically significant interactions.

The population structure of the MAVAN, GUSTO and ALSPAC cohorts were evaluated using principal component analysis of all autosomal SNPs that passed the quality control without low allele frequency (MAF > 5%) and that were not in high linkage disequilibrium (*r*^2^ > 0.2) across 50 kb regions, and a sliding window of 5 SNPs for MAVAN and GUSTO cohorts, and not in high linkage disequilibrium across 100-kilobase region, an increment of 5 SNPs, and variance inflation factor threshold of 1.01 for ALSPAC cohort. Based on the inspection of the scree plot, the first principal components were the most informative concerning population structure in the cohorts and were included in all subsequent analyses^76,77^. Supplementary Figs. 4–6.

Differences in means on the main confounding variables of the study were tested using Student’s t-test for categorical variables or correlations test for continuous variables. No differences were found in regarding to the main confounding variables in both cohorts (Supplementary Tables 4–6). A descriptive statistic for the domains of the CEBQ questionnaire in the cohorts are reported in the Supplementary material.

### Reporting summary

Further information on research design is available in the Nature Research Reporting Summary linked to this article.

## Supplementary information

Supplementary information

Description of Additional Supplementary Files

Supplementary Data 1

Supplementary Data 2

Supplementary Data 3

Supplementary Data 4

Reporting Summary

## Data Availability

Data from MAVAN can be made available upon request to the corresponding author, due to ethical restrictions. The original contributions presented in the study are included in the article/Supplementary material, further inquiries can be directed to the corresponding author. A project description and scientific reasoning will be needed for data request. Data From GUSTO can be requested at (https://gustodatavault.sg/). Data from ALSPAC can be requested at (http://www.bristol.ac.uk/alspac/researchers/our-data/).

## Code availability

The pipeline for ePRS calculation is available on: https://github.com/SilveiraLab/LepR-ePRS. Custom code is available on 10.5281/zenodo.7041982.
