# Supplementary material for: Leptin receptor co-expression gene network moderates the effect of early life adversity on eating behavior in children
Source: Commun Biol. 2022 Oct 14;5:1092. doi: 10.1038/s42003-022-03992-8 (PMC9568584; doi:10.1038/s42003-022-03992-8)
Supplement: Supplementary file 1 — Supplementary information [file 42003_2022_3992_MOESM1_ESM.pdf]

Contents

Supplementary Figure 1: Main effect of Prefrontal-based LepR-ePRS on CEBQ slowness score. 2

Supplementary Figure 2: Leptin receptor gene network in the hypothalamus. 3

Supplementary Figure 3: Enrichment analysis of the Leptin receptor gene network in the prefrontal cortex. 4

Supplementary Figure 4: Scree Plots and of the top 25 PCs from MAVAN cohort. 5

Supplementary Figure 5: Scree Plots and of the top 25 PCs from GUSTO cohort. 6

Supplementary Figure 6: Scree Plots and of the top 25 PCs from ALSPAC cohort. 7

Supplementary Table 1: 8

Supplementary Table 2: 9

Supplementary Table 3 10

Supplementary Table 4: 11

Supplementary Table 5: 12

Supplementary Table 6: 13

Supplementary Figure 1: Main effect of Prefrontal-based LepR-ePRS on CEBQ slowness score: at (A) 48 and (B) 72 months. MAVAN cohort (48m N=133, 72m N=129).

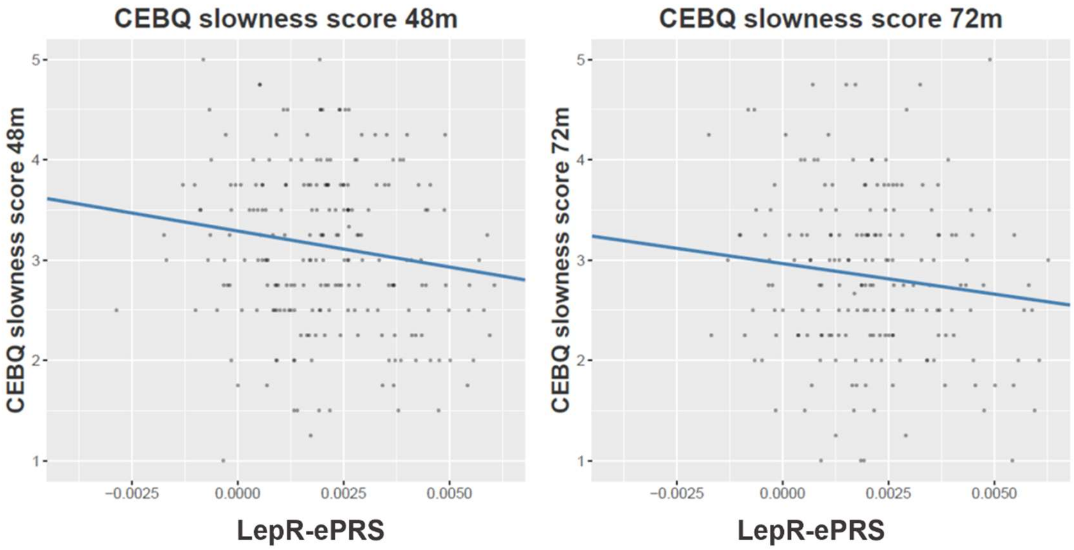

Supplementary Figure 2: Leptin receptor gene network in the hypothalamus. Node size represents the InDegree (connections between the protein with other proteins); Node border thickness represents OutDegre (connections of other proteins with the target protein); Edge thickness represents co-expression between genes.

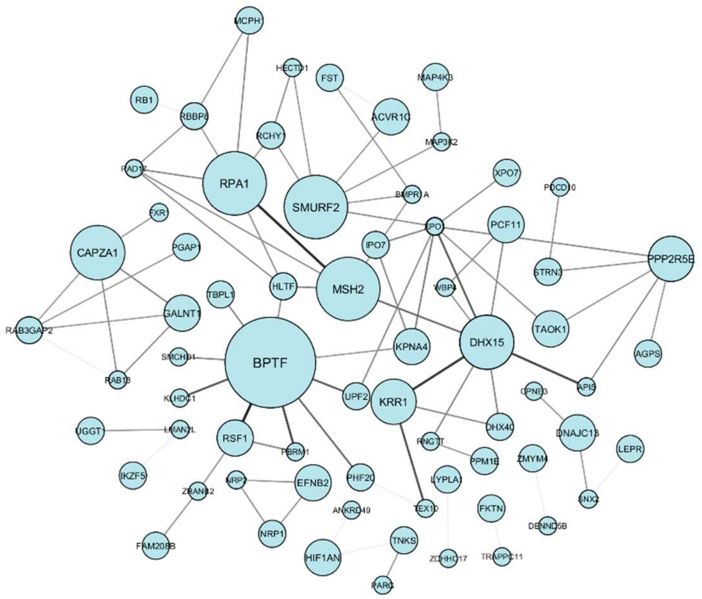

Supplementary Figure 3: Enrichment analysis of the Leptin receptor gene network in the prefrontal cortex. (a) Genes co-expressed with the LepR in the prefrontal cortex have enriched expression during fetal and early childhood development of the amygdala, cortex, and thalamus (CSEA tool). (b) Synapse ontology showing that LEPR network in the prefrontal cortex is enriched for synaptic components, such as synapse (UBA52 RPS3 RPS25 RPL7), postsynapse (CYFIP1), regulation of synapse organization (CAMK1), among others (SynGO). (c) Biological processes associated with LepR network, genes are enriched for cellular process (82 genes), biological regulation (45 genes), cellular component organization or biogenesis (31 genes), metabolic process (51 genes), and more. (d) Molecular function associated with LepR network, to genes were enriched binding function (56 genes), catalytic activity (35 genes), and molecular function regulator (7 genes). (e) DYRK2 is the only common gene between the gene networks (List 1 PFC and List 2: hypothalamus).

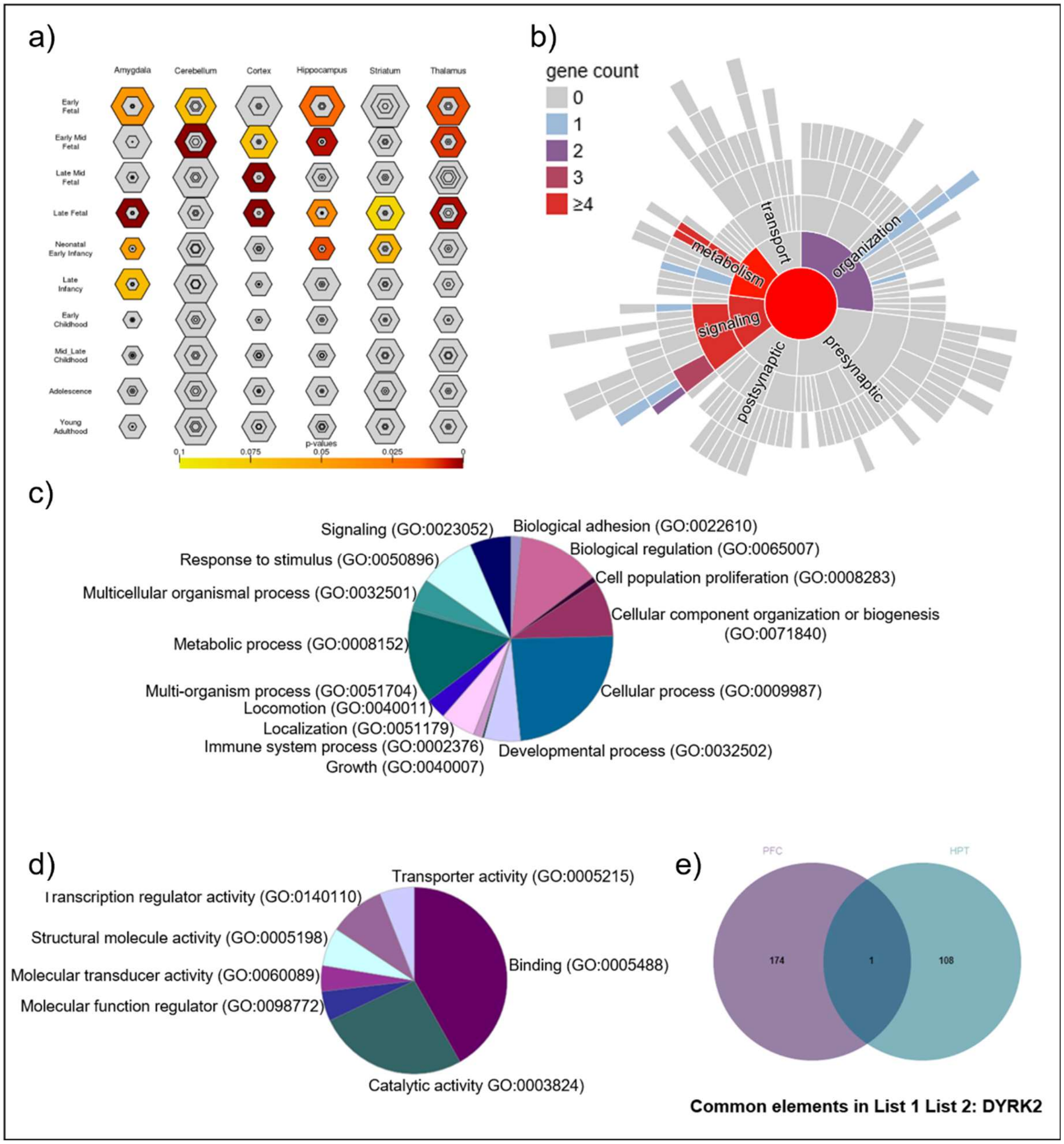

Supplementary Figure 4: Scree Plots and of the top 25 PCs from MAVAN cohort.

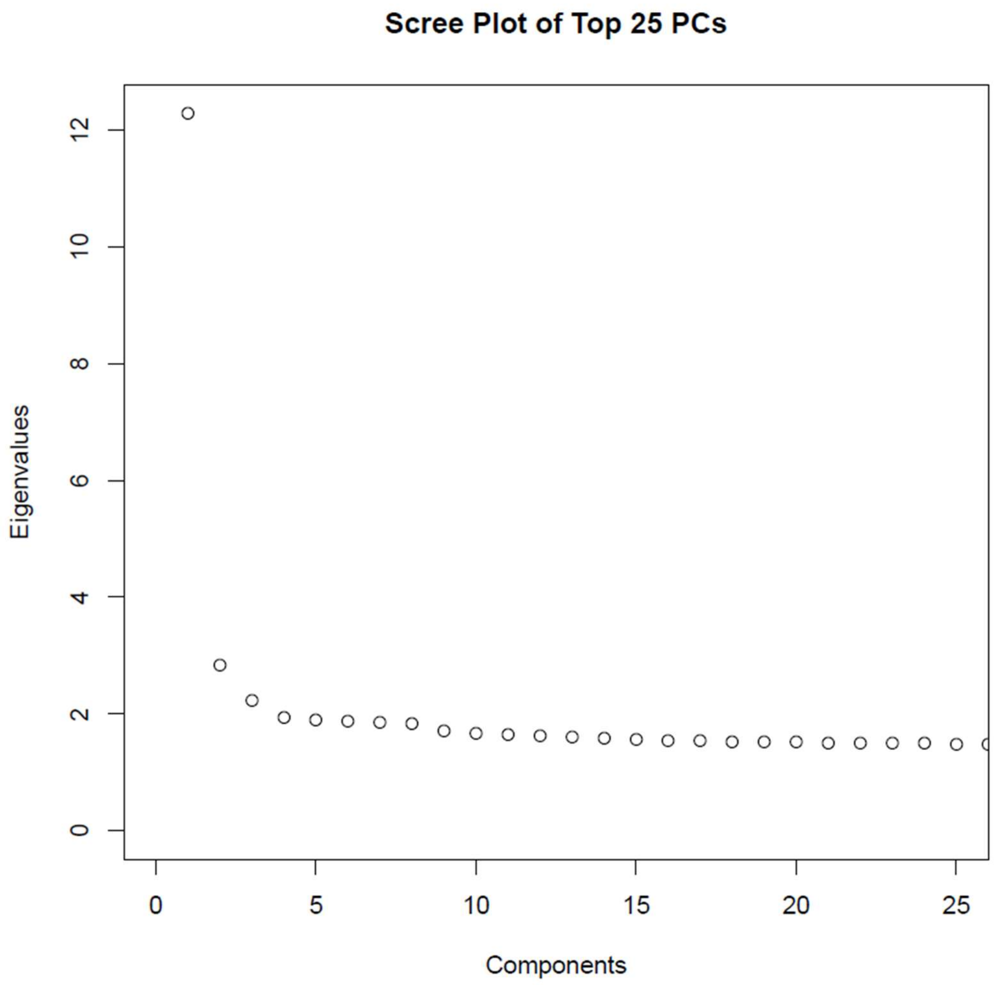

Supplementary Figure 5: Scree Plots and of the top 25 PCs from GUSTO cohort.

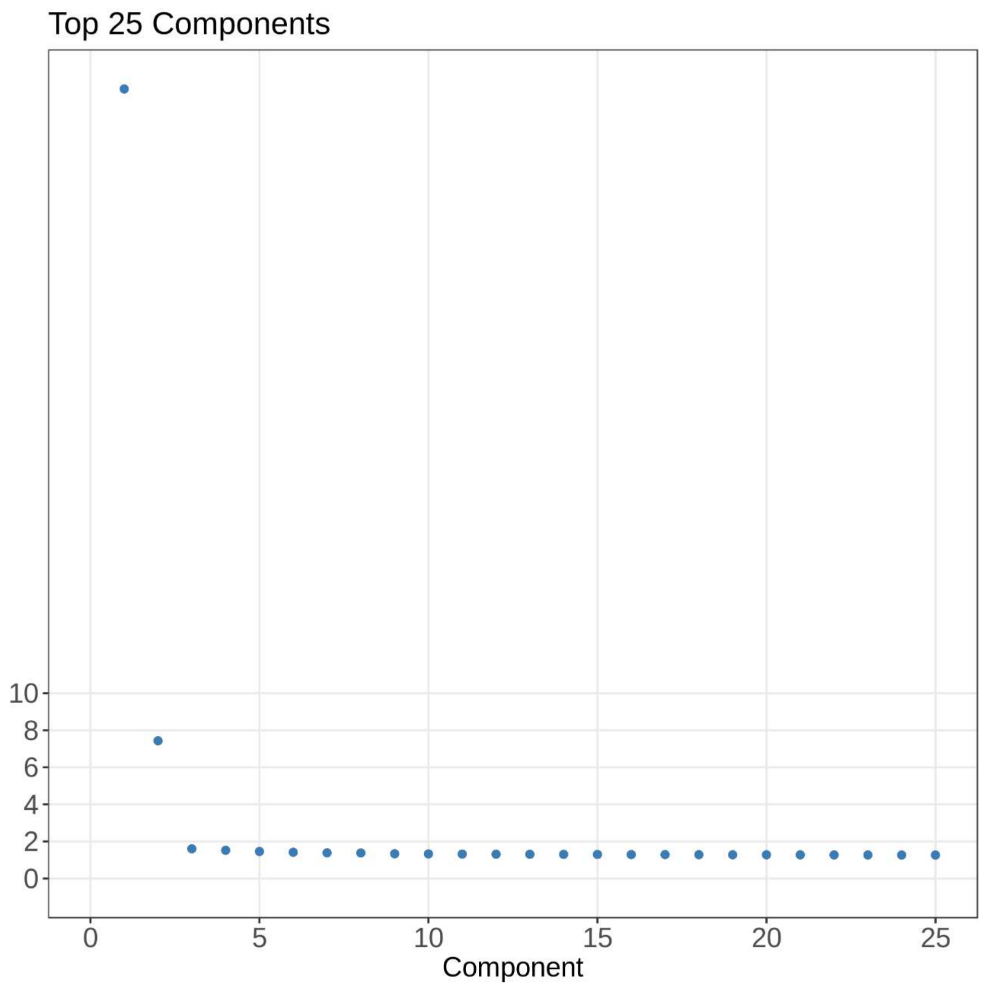

Supplementary Figure 6: Scree Plots and of the top 25 PCs from ALSPAC cohort.

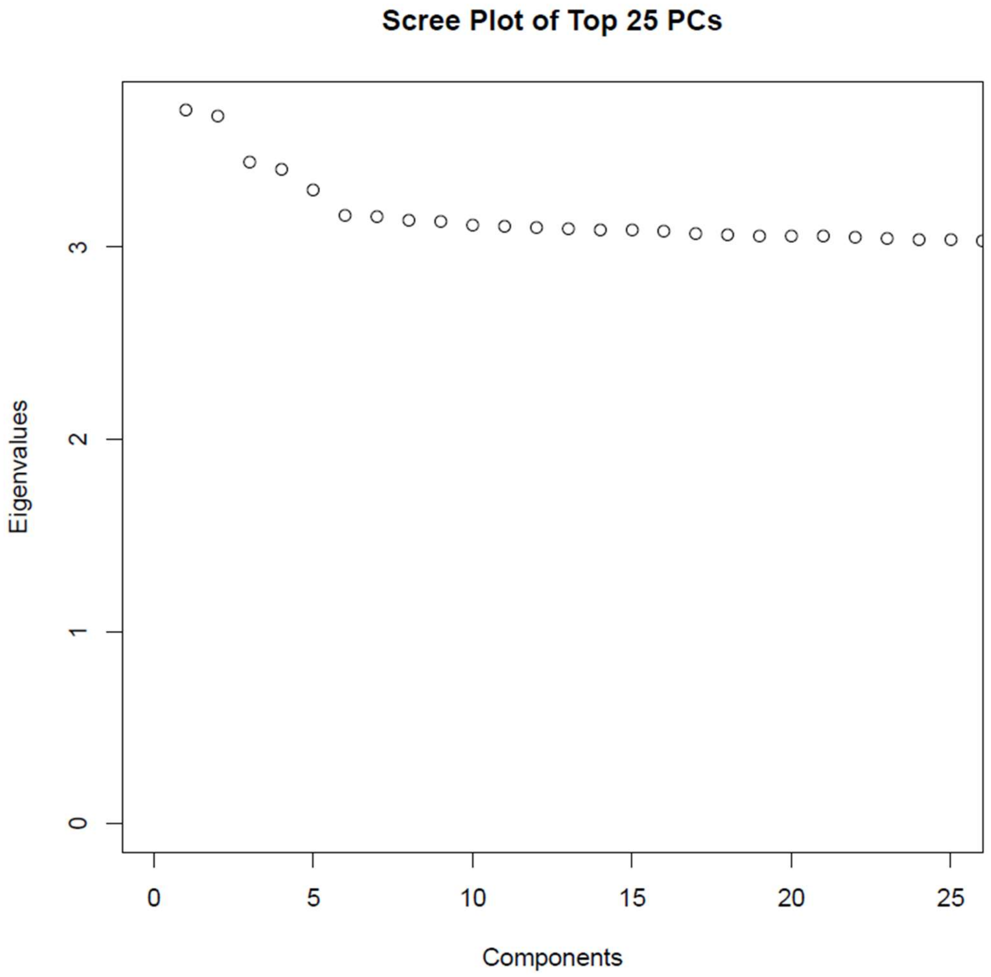

Supplementary Table 1: ePRS and ePRS x Adversity effects on the CEBQ outcomes in MAVAN cohort using prefrontal cortex (PFC) and hypothalamus (HPT) ePRS-LepR.

			**MAVAN**		
Outcome	*Adversity*	*LepR-ePRS PFC*	*Adversity and LepR-ePRS PFC*	*LepR-ePRS HPT*	*Adversity and LepR-HPT*
Desire to drink score 48m	β=0·21, p=0·00006**<br>CI 0·11 — 0·31	β=-14·37, p=0·73<br>CI -98·15 — 69·40	β=33·92, p=0·33<br>CI -34·34 — 102·18	β=-14·85, p=0·62<br>CI -74·61 — 44·93	β=-22·28, p=0·38<br>CI -72·9 — 28·31
Desire to drink score 72m	β=0·14, p=0·004*<br>CI 0·05 — 0·23	β=-19·74, p=0·65<br>CI -105·68 — 66·18	β=-11·55, p=0·72<br>CI -76·27 — 53·16	β=-15·11, p=0·63<br>CI -77·07 — 46·85	β=-38·88, p=0·10<br>CI -84·90 — 7·12
Emotional overeating score 48m	β=0·05, p=0·09<br>CI -0·01 — 0·12	β=46·45, p=0·052<br>CI -0·18 — 93·09	β=31·94, p=0·11<br>CI -7·96 — 71·85	β=-14·46, p=0·40<br>CI -47·98 — 19·06	β=-5·51, p=0·71<br>CI -35·55 —24·52
Emotional overeating score 72m	β=0·06, p=0·06<br>CI -0·003 — 0·13	β=45·12, p=0·12<br>CI -11·50 — 101·75	β=2·95, p=0·89<br>CI -42·26 — 48·16	β=-18·86, p=0·36<br>CI -59·88 — 22·15	β=0·87, p=0·95<br>CI -31·97 — 33·72
Emotional undereating score 48m	β=-0·01, p=0·8<br>CI -0·08 — 0·10	β=-2·47, p=0·94<br>CI -73·16 — 68·21	β=-4·13, p=0·89<br>CI -62·77 — 54·51	β=-16·60, p=0·51<br>CI -66·98 — 33·80	β=-4·53, p=0·83<br>CI -47·99 — 38·93
Emotion undereating score 72m	β=0·05, p=0·30<br>CI -0·04 — 0·13	β=-18·00, p=0·63<br>CI -92·83 — 56·82	β=-16·84, p=0·58<br>CI -77·08 — 43·4	β=-26·41, p=0·33<br>CI -80·27 — 27·44	β=4·79, p=0·82<br>CI -38·42 — 48·00
Enjoyment score 48m	β=-0·08, p=0·03*<br>CI -0·15 — -0·005	β=17·59, p=0·56<br>CI -42·36 — 77·55	β=61·58, p=0·015*<br>CI 12·71 — 110·44	β=8·40, p=0·70<br>CI -34·42 — 51·19	β=14·43, p=0·44<br>CI -22·7 —51·56
Enjoyment score 72m	β=-0·05, p=0·21<br>CI -0·13 — 0·03	β=26·10, p=0·45<br>CI -42·23 — 94·45	β=97·78, p=0·001**<br>CI 39·4 — 156·17	β=-40·9, p=0·10<br>CI -89·91 — 8·08	β=9·85, p=0·62<br>CI -29·91 — 49·62
Fussiness score 48m	β=0·03, p=0·46<br>CI -0·05 — 0·12	β=-9·33, p=0·78<br>CI -76·20 — 57·53	β=-52·94, p=0·06<br>CI -108·6 — 2·7	β=-13·60, p=0·60<br>CI -61·26 — 34·12	β=-35·27, p=0·09<br>CI -76·77 — 6·22
Fussiness score 72m	β=0·03, p=0·44<br>CI -0·05 — 0·12	β=-32·04, p=0·41<br>CI -109·00 — 44·91	β=-47·45, p=0·11<br>CI -106·5 — 11·55	β=20·35, p=0·50<br>CI -35·16 — 75·87	β=-40·45, p=0·06<br>CI -83·02 — 2·11
Responsiveness score 48m	β=0·02, p=0·64<br>CI -0·06 — 0·10	β=29·13, p=0·35<br>CI -32·79 — 91·06	β=83·79, p=0·009*<br>CI 21·6 — 146·01	β=2·26, p=0·92<br>CI -42·01 — 46·55	β=-24·20, p=0·25<br>CI -65·98 — 17·57
Responsiveness score 72m	β=0·06, p=0·20<br>CI -0·03 — 0·15	β=28·11, p=0·47<br>CI -48·66 — 104·89	β=40·55, p=0·18<br>CI -19·5 — 100·6	β=-0·57, p=0·98<br>CI -56·02 — 54·87	β=-6·20, p=0·78<br>CI -49·96 — 37·56
Satiety score 48m	β=0·02, p=0·52<br>CI -0·04 — 0·08	β=-13·50, p=0·61<br>CI -65·70 — 38·71	β=-43·63, p=0·047*<br>CI -86·22 — -1·04	β=15·15, p=0·42<br>CI -22·06 — 52·38	β=-1·06, p=0·94<br>CI-33·24 — 31·12
Satiety score 72m	β=-0·01, p=0·74<br>CI -0·08 — 0·06	β=-37·21, p=0·23<br>CI -98·12 — 23·68	β=-41·15, p=0·094<br>CI -88·91 — 6·6	β=-0·52, p=0·99<br>CI -44·61 — 43·57	β=-21·94, p=0·22<br>CI -56·94 — 13·06
Slowness score 48m	β=0·05, p=0·21<br>CI -0·03 — 0·13	β=-71·89, p=0·02*<br>CI -134·90 — -8·89	β=-39·69, p=0·132<br>CI -90·99 — 11·61	β=30·93, p=0·20<br>CI -14·36 — 76·23	β=4·25, p=0·83<br>CI -34·70 — 43·21
Slowness score 72m	β=0·01, p=0·80<br>CI -0·07 — 0·09	β=-60·92, p=0·09<br>CI -132·03 — 10·18	β=-34·69, p=0·208<br>CI -88·43 — 19·04	β=42·20, p=0·10<br>CI -9·11 — 93·50	β=-10·567, p=0·60<br>CI -50·36 — 29·22

* Significant effect (p<0·05).

** Significant effect after correction for multiple testing (Bonferroni-Holm procedure applied).

CI: 95% confidence interval.

Supplementary Table 2: Linear regression effects on the CEBQ outcomes in GUSTO Cohort using prefrontal cortex (PFC) and hypothalamus (HPT) ePRS-LepR.

			GUSTO		
Outcome	*Adversity*	*LepR-ePRS PFC*	*Adversity and LepR-ePRS PFC*	*LepR-ePRS HPT*	*Adversity and LepR-ePRS HPT*
Desire to drink score 60m	β=0·09, p=0·02*<br>CI 0·02 — 0·18	β=-3·56, p=0·72<br>CI -23·64 — 16·51	β=-3·83, p=0·75<br>CI -28·23 — 20·57	β=-2·81, p=0·66<br>CI -15·53 — 9·89	β=-1·73, p=0·82<br>CI -17·32 — 13·85
Emotional overeating score 60m	β=-0·0072. p=0·97<br>CI -0·05 — 0·05	β=-6·98, p=0·30<br>CI -20·07 — 6·09	β=-3·14, p=0·68<br>CI -18·50 — 12·23	β=0·74, p=0·85<br>CI -7·54 — 9·03	β=-5·62, p=0·26<br>CI -15·5 — 4·25
Emotional undereating score 60m	β=-0·10, p=0·003**<br>CI -0·18 — -0·04	β=-11·32, p=0·20<br>CI -28·98 — 6·32	β=-2·36, p=0·82<br>CI -23·5 — 18·75	β=2·95, p=0·60<br>CI -8·22 — 14·14	β=-8·76, p=0·20<br>CI -22·25 — 4·74
Enjoyment score 60m	β=-0·02, p=0·51<br>CI -0·05 — 0·10	β=3·20, p=0·72<br>CI -14·63 — 21·04	β=30·48, p=0·006*<br>CI 8·65 — 52·32	β=-3·62, p=0·52<br>CI -14·92 — 7·66	β =-5·64, p=0·43<br>CI -19·75 — 8·5
Fussiness score 60m	β=-0·05, p=0·09<br>CI -0·12 — 0·01	β=-4·86, p=0·56<br>CI -21·52 — 11·79	β=-24·07, p=0·02*<br>CI -44·37 — -3·77	β=-0·95, p=0·85<br>CI -11·51 — 9·59	β=6·28, p=0·33<br>CI -6·66 — 19·52
Responsiveness score 60m	β=0·03, p=0·27<br>CI -0·03 — 0·10	β=-9·24, p=0·25<br>CI -25·08 — 6·58	β=-3·47, p=0·71<br>CI -22·16 — 15·22	β=-2·32, p=0·65<br>CI -12·35 — 7·70	β=-3·92, p=0·52<br>CI -15·91 — 8·06
Satiety score 60m	β=-0·07, p=0·004*<br>CI -0·13 — -0·02	β=-1·10, p=0·87<br>CI -14·52 — 12·31	β=-17·0, p=0·03*<br>CI -32·97 — -1·03	β=0·64, p=0·88<br>CI -7·85 — 9·14	β=-2·12, p=0·68<br>CI -12·4 — 8·15
Slowness score 60m	β=-0·06, p=0·079<br>CI -0·13 — 0·01	β=0·97, p=0·91<br>CI -17·04 — 18·99	β=-11·24, p=0·30<br>CI-32·66 — 10·17	β=-5·12, p=0·37<br>CI -16·52 — 6·27	β=-4·54, p=0·51<br>CI -18·30 — 9·20

* Significant effect (p<0·05).

** Significant effect after correction for multiple testing (Bonferroni-Holm procedure applied).

CI: 95% confidence interval.

Supplementary Table 3: Enrichment analysis for gene ontology processes of the LepR co-expressed genes in the prefrontal cortex using MetaCore.

Processes	*P-value*	*FDR (false discovery rate)*
Cellular response to oxygen-containing compound	8·741E-14	3·355E-10
Regulation of multicellular organismal process	2·582E-12	3·127E-09
Response to endogenous stimulus	2·834E-12	3·127E-09
Circulatory system development	3·693E-12	3·190E-09
Cellular response to chemical stimulus	4·047E-12	3·190E-09
Negative regulation of angiogenesis	6·689E-12	3·836E-09
Regulation of cellular component movement	6·942E-12	3·836E-09
Regulation of cell migration	6·952E-12	3·836E-09
Nervous system development	1·032E-11	4·441E-09
Negative regulation of multicellular organismal process	1·059E-11	4·441E-09
Response to oxygen-containing compound	1·127E-11	4·441E-09
System development	1·304E-11	4·795E-09
Response to wounding	2·831E-11	7·811E-09
Cardiovascular system development	3·609E-11	8·928E-09
Cellular response to organic substance	3·721E-11	8·928E-09
Regulation of cell motility	4·037E-11	9·281E-09
Response to cAMP	4·339E-11	9·578E-09
Response to hypoxia	4·762E-11	1·006E-08
Negative regulation of biological process	4·922E-11	1·006E-08
Multicellular organism development	7·258E-11	1·430E-08
Regulation of endothelial cell migration	8·263E-11	1·553E-08
Anatomical structure morphogenesis	8·441E-11	1·553E-08
Response to decreased oxygen levels	1·042E-10	1·639E-08
Response to hormone	1·043E-10	1·639E-08
Developmental process	1·073E-10	1·639E-08
Anatomical structure formation involved in morphogenesis	1·090E-10	1·639E-08
Positive regulation of multicellular organismal process	1·096E-10	1·639E-08
Anatomical structure development	1·099E-10	1·639E-08
Response to peptide	7·385E-10	6·907E-08
Response to lipid	1·064E-09	9·322E-08
Neurogenesis	7·847E-09	4·656E-07
Response to insulin	1·526E-08	8·095E-07
Regulation of neuron differentiation	2·544E-08	1·200E-06
Response to food	8·351E-04	4·562E-03
Response to leptin	2·909E-03	1·194E-02
Regulation of lipid localization	3·960E-03	1·519E-02

Supplementary Table 4: Participants' characteristics in MAVAN. Numbers are presented as mean (SD) or percentage (number of participants). Differences in N are due to missing values.

Variable	*Groups: Mean (SD, N)*		*F*	*P*
Sex	Male	Female		
	ePRS-LepR PFC: 0·002 (0·001, N=72)	ePRS-LepR PFC: 0·002 (0·002, N=71)	0·34	0·56
	ePRS-LepR HPT: -0·002 (0·002, N=72)	ePRS-LepR HPT: -0·002 (0·002, N=71)	1·4	0·23
Smoking during pregnancy	Yes	No		
	ePRS-LepR PFC: 0·002 (0·002, N=17)	ePRS-LepR PFC: 0·002 (0·001, N=126)	0·014	0·90
	ePRS-LepR HPT: -0·002 (0·002, N=17)	ePRS-LepR HPT: -0·002 (0·002, N=126)	0·53	0·46
Maternal education	University degree or above	College or lower		
	ePRS-LepR PFC: 0·002 (0·001, N=83)	ePRS-LepR PFC: 0·002 (0·002, N=60)	0·001	0·98
	ePRS-LepR HPT: -0·002 (0·002, N=83)	ePRS-LepR HPT: -0·002 (0·002, N=60)	1·52	0·22
Income	Low	High		
	ePRS-LepR PFC: 0·002 (0·002, N=20)	ePRS-LepR PFC: 0·002 (0·001, N=110)	2·37	0·12
	ePRS-LepR HPT: -0·002 (0·002, N=20)	ePRS-LepR HPT: -0·002 (0·002, N=110)	0·12	0·73
Variables	*Pearson Correlation*		*N*	*P*
Age of mother at Birth	ePRS-LepR PFC: 0·083		143	0·32
	ePRS-LepR HPT: 0·028		143	0·73
Gestational age: weeks	ePRS-LepR PFC: 0·036		143	0·66
	ePRS-LepR HPT: -0·053		143	0·52
Birth weight grams	ePRS-LepR PFC: 0·74		143	0·38
	ePRS-LepR HPT: -0·06		143	0·47
Breastfeeding in months	ePRS-LepR PFC: 0·61		143	0·47
	ePRS-LepR HPT: -0·166		143	0·051
CEBQ (48m N=133, 72m N=129)			Mean	SD
Responsiveness score 48m			2·92	0·84
Emotional overeating score 48m			1·61	0·61
Enjoyment score 48m			3·57	0·76
Desire to drink score 48m			3·06	1·1
Satiety score 48m			3·17	0·66
Slowness score 48m			3·12	0·79
Emotional undereating score 48m			3·05	0·88
Fussiness score 48m			3·03	0·84
Responsiveness score 48m			2·26	0·89
Emotional overeating score 72m			1·68	0·68
Enjoyment score 72m			3·56	0·83
Desire to drink score 72m			2·57	0·99
Satiety score 72m			3·07	0·72
Slowness score 48m			2·91	0·83
Emotional undereating score 72m			2·77	0·89
Fussiness score 72m			3·05	0·89

Supplementary Table 5: Participants' characteristics in GUSTO. Numbers are presented as mean (SD) or percentage (number of participants). Differences in N are due to missing values.

Variables	*Groups: Mean (SD, N)*		*F*	*P*
Sex	Male	Female		
	ePRS-LepR PFC: 0·002 (0·003, N=233)	ePRS-LepR PFC: 0·002 (0·003, N=206)	2·33	0·13
	ePRS-LepR HPT: -0·002 (0·005, N=233)	ePRS-LepR HPT: -0·002 (0·004, N=206)	0·007	0·93
Smoking during pregnancy	Yes	No		
	ePRS-LepR PFC: 0·003 (0·003, N=10)	ePRS-LepR PFC: 0·002 (0·003, N=429)	0·07	0·78
	ePRS-LepR HPT: -0·003 (0·004, N=10)	ePRS-LepR HPT: -0·002 (0·005, N=429)	0·22	0·63
Breastfeeding for at least 3 months	< 3 m	≥ 3 m		
	ePRS-LepR PFC: 0·002 (0·003, N=174)	ePRS-LepR PFC: 0·002 (0·003, N=257)	0·33	0·56
	ePRS-LepR HPT: -0·002 (0·004, N=174)	ePRS-LepR HPT: -0·002 (0·005, N=257)	2·30	0·13
Variables	*Pearson Correlation*		*N*	*P*
Age of mother at birth	ePRS-LepR PFC: 0·007		439	0·88
	ePRS-LepR HPT: -0·01		439	0·83
Gestational age: weeks	ePRS-LepR PFC: 0·02		439	0·70
	ePRS-LepR HPT: -0·04		439	0·42
Birth weight grams	ePRS-LepR PFC: 0·04		439	0·35
	ePRS-LepR HPT: 0·04		439	0·44
CEBQ (N=439)			Mean	SD
Responsiveness score 60m			2.40	0.68
Emotional overeating score 60m			1.79	0.56
Enjoyment score 60m			3.49	0.79
Desire to drink score 60m			2.73	0.89
Satiety score 60m			2.87	0.59
Slowness score 60m			2.96	0.79
Emotional undereating score 60m			2.79	0.79
Fussiness score 60m			3.00	0.73

Supplementary Table 6: Participants' characteristics in ALSPAC. Numbers are presented as mean (SD) or percentage (number of participants). Differences in N are due to missing values.

Variables	*Groups: Mean (SD, N)*		*F*	*P*
Sex	Male	Female		
	ePRS-LepR PFC: 2·86 (8·97, N=1915)	ePRS-LepR PFC: 2·83 (9·50, N=1839)	6·21	0·013
	ePRS-LepR HPT: -0·004 (0·001, N= 1915)	ePRS-LepR HPT: -0·004 (0·001, N=206)	3·62	0·06
Highest maternal education	Low	High		
	ePRS-LepR PFC: 2·84 (9·25e-04, N=2857)	ePRS-LepR PFC: 2·90 (9·30e-04, N=696)	0·41	0·52
	ePRS-LepR HPT: -0·004 (0·001, N=2857)	ePRS-LepR HPT: -0·004 (0·001, N=696)	0·09	0·76
Crowding index 2y9m	< 0·75	≥ 0·75		
	ePRS-LepR PFC: 2·83 (9·30e-04, N=2429)	ePRS-LepR PFC: 2·87 (9·13e-04, N=1325)	0·33	0·56
	ePRS-LepR HPT: -0·004 (0·001, N=2429)	ePRS-LepR HPT: -0·004 (0·001, N=1325)	2·30	0·13
Breastfeeding for at least 3 months	< 3 m	≥ 3 m		
	ePRS-LepR PFC: 2·88 (9·13e-04, N=1648)	ePRS-LepR PFC: 2·84 (9·33e-04, N=1850)	0·31	0·57
	ePRS-LepR HPT: -0·004 (0·001, N=1648)	ePRS-LepR HPT: -0·004 (0·001, N=1850)	0·40	0·52
Variables	*Pearson Correlation*		*N*	*P*
Gestational age: weeks	ePRS-LepR PFC: 0·008		3754	0·61
	ePRS-LepR HPT: 0·01		3754	0·50
Age of mother at Birth	ePRS-LepR PFC: -0·008		3754	0·61
	ePRS-LepR HPT: 0·02		3754	0·23
Birth weight	ePRS-LepR PFC: 0·017		3754	0·29
	ePRS-LepR HPT: -0·002		3754	0·90
Metabolic *Variables*			Mean	SD
Log Leptin	N=3544		1.79	0.77
Glucose	N=688		4.95	0.39
