# Supplementary material for: Leptin receptor co-expression gene network moderates the effect of early life adversity on eating behavior in children
Source: Commun Biol. 2022 Oct 14;5:1092. doi: 10.1038/s42003-022-03992-8 (PMC9568584; doi:10.1038/s42003-022-03992-8)
Supplement: Supplementary file 2 — Description of Additional Supplementary Files [file 42003_2022_3992_MOESM2_ESM.pdf]

Description of Additional Supplementary Files

File name: Supplementary Data 1
Description: Final list of genes included in the PCF ePRS.

File name: Supplementary Data 2
Description: Final list of genes included in the HPT ePRS.

File name: Supplementary Data 3
Description: Numerical data used to create figures.

File name: Supplementary Data 4
Description: Complete list of SNPs used to calculate the ePRS in each cohort.
