# Supplementary material for: Leptin receptor co-expression gene network moderates the effect of early life adversity on eating behavior in children
Source: Commun Biol. 2022 Oct 14;5:1092. doi: 10.1038/s42003-022-03992-8 (PMC9568584; doi:10.1038/s42003-022-03992-8)
Supplement: Supplementary file 7 — Reporting Summary [file 42003_2022_3992_MOESM7_ESM.pdf]

Corresponding author(s): Patrícia Pelufo Silveira
Last updated by author(s): May 24, 2022

Reporting Summary

Nature Portfolio wishes to improve the reproducibility of the work that we publish. This form provides structure for consistency and transparency in reporting. For further information on Nature Portfolio policies, see our Editorial Policies and the Editorial Policy Checklist.

Statistics

For all statistical analyses, confirm that the following items are present in the figure legend, table legend, main text, or Methods section.

n/a	Confirmed	
☐	☒	The exact sample size (*n*) for each experimental group/condition, given as a discrete number and unit of measurement
☐	☒	A statement on whether measurements were taken from distinct samples or whether the same sample was measured repeatedly
☐	☒	The statistical test(s) used AND whether they are one- or two-sided<br>*Only common tests should be described solely by name; describe more complex techniques in the Methods section.*
☐	☒	A description of all covariates tested
☐	☒	A description of any assumptions or corrections, such as tests of normality and adjustment for multiple comparisons
☐	☒	A full description of the statistical parameters including central tendency (e.g. means) or other basic estimates (e.g. regression coefficient) AND variation (e.g. standard deviation) or associated estimates of uncertainty (e.g. confidence intervals)
☐	☒	For null hypothesis testing, the test statistic (e.g. *F*, *t*, *r*) with confidence intervals, effect sizes, degrees of freedom and *P* value noted<br>*Give P values as exact values whenever suitable.*
☒	☐	For Bayesian analysis, information on the choice of priors and Markov chain Monte Carlo settings
☒	☐	For hierarchical and complex designs, identification of the appropriate level for tests and full reporting of outcomes
☒	☐	Estimates of effect sizes (e.g. Cohen's *d*, Pearson's *r*), indicating how they were calculated

Our web collection on statistics for biologists contains articles on many of the points above.

Software and code

Policy information about availability of computer code

Data collection	Not applicable
Data analysis	Data analysis was conducted using R Studio (version 1.4.1717).

For manuscripts utilizing custom algorithms or software that are central to the research but not yet described in published literature, software must be made available to editors and reviewers. We strongly encourage code deposition in a community repository (e.g. GitHub). See the Nature Portfolio guidelines for submitting code & software for further information.

Data

Policy information about availability of data

All manuscripts must include a data availability statement. This statement should provide the following information, where applicable:

- Accession codes, unique identifiers, or web links for publicly available datasets
- A description of any restrictions on data availability
- For clinical datasets or third party data, please ensure that the statement adheres to our policy

Data related to this study (MAVAN and GUSTO cohorts) will be made available upon reasonable request to the corresponding author. ALSPAC data has to be requested directly from ALSPAC.

Human research participants

Policy information about studies involving human research participants and Sex and Gender in Research.

Reporting on sex and gender	Sex as a biological attribute was used as a covariate for all analyses of the study.
Population characteristics	See below (Behavioural & social sciences study design)
Recruitment	MAVAN and GUSTO: pregnant women aged 18 years and above were recruited at research affiliated hospitals and mother-child dyads were followed over childhood. ALSPAC: pregnant women living in the county of Avon, UK, with delivery date between April 1, 1991 and December 31, 1992.
Ethics oversight	Approval for the MAVAN project was obtained from obstetricians performing deliveries at the study hospitals and by the ethics committees and university affiliates (McGill University and Université de Montréal, the Royal Victoria Hospital, Jewish General Hospital, Centre hospitalier de l'Université de Montréal and Hôpital Maisonneuve-Rosemont) and St Joseph's Hospital and McMaster University, Hamilton, Ontario, Canada. Approval for the GUSTO cohort was obtained from National Healthcare Group Domain Specific Review Board and the SingHealth Centralised Institutional Review Board. Ethical approval of the ALSPAC study was obtained from the Ethics and Law Committee and Local Research Ethics Committees. A full list of the ethics committees that approved different aspects of the ALSPAC studies is available at http://www.bristol.ac.uk/alspac/researchers/research-ethics/.

Note that full information on the approval of the study protocol must also be provided in the manuscript.

Field-specific reporting

Please select the one below that is the best fit for your research. If you are not sure, read the appropriate sections before making your selection.

☐ Life sciences ☒ Behavioural & social sciences ☐ Ecological, evolutionary & environmental sciences

For a reference copy of the document with all sections, see nature.com/documents/nr-reporting-summary-flat.pdf

Behavioural & social sciences study design

All studies must disclose on these points even when the disclosure is negative.

Study description	Cross-sectional analyses of longitudinal birth cohorts
Research sample	Three prospective observational birth cohorts. The Canadian sample (the Maternal Adversity, Vulnerability and Neurodevelopment Project, MAVAN) [REF: O'Donnell et al., 2014] included children of 48 to 72 months old from Montreal (Quebec) and Hamilton (Ontario). The Singaporean sample (the Growing Up in Singapore Towards healthy Outcomes, GUSTO) [REF: Soh et al., 2014] comprised of 60 months old children. The British sample was derived from The Avon Longitudinal Study of Parents and Children (ALSPAC) [REF: Boyd et al., 2013; Fraser et al., 2013; Northstone et al., 2019] and comprised of children 8.5 and 9.5 years old.
Sampling strategy	The sampling strategy involved voluntary participation of pregnant women according to the studies eligibility criteria. Sample sizes of the data sets used in this study were not predetermined but subject to available behavioral, genetic, and postnatal environmental data.
Data collection	Data collection was performed in different forms: in-person using pen and paper questionnaires, self reports, computer assessments. Genetic samples were collected via buccal saliva samples or blood samples.
Timing	Data collection started at the gestational period and participants were followed-up until preadolescence in MAVAN and GUSTO and until young adulthood in ALSPAC.
Data exclusions	We applied the recruitment criteria of MAVAN and GUSTO to ALSPAC cohort to make the cohorts comparable, which reduced ALSPAC cohort to 12506 participants. For the analyses we considered all cases with complete data available.
Non-participation	Attrition was present in all three cohorts since this is a common feature of longitudinal studies. MAVAN attrition rate was 38.3-52%, GUSTO - 26.1% and ALSPAC - 4.2% (taking into account additional recruitment). Further details on the attrition rates and reasons can be found in the reference papers.
Randomization	Not applicable.

Reporting for specific materials, systems and methods

We require information from authors about some types of materials, experimental systems and methods used in many studies. Here, indicate whether each material, system or method listed is relevant to your study. If you are not sure if a list item applies to your research, read the appropriate section before selecting a response.

Materials & experimental systems

n/a	Involved in the study
☒	☐ Antibodies
☒	☐ Eukaryotic cell lines
☒	☐ Palaeontology and archaeology
☒	☐ Animals and other organisms
☒	☐ Clinical data
☒	☐ Dual use research of concern

Methods

n/a	Involved in the study
☒	☐ ChIP-seq
☒	☐ Flow cytometry
☒	☐ MRI-based neuroimaging
